# Social determinants of health rather than race impact health-related quality of life in 10-year-old children born extremely preterm

**DOI:** 10.3389/fped.2024.1359270

**Published:** 2024-03-14

**Authors:** Catherine Call, Ali Oran, T. Michael O’Shea, Elizabeth T. Jensen, Jean A. Frazier, Ruben Vaidya, Jeffrey Shenberger, Semsa Gogcu, Michael E. Msall, Sohye Kim, Isha Jalnapurkar, Rebecca C. Fry, Rachana Singh

**Affiliations:** ^1^ Department of Pediatrics, Tufts University School of Medicine, Boston, MA, United States; ^2^ Department of Environmental Sciences & Engineering, Gillings School of Public Health, The University of North Carolina at Chapel Hill, Chapel Hill, NC, United States; ^3^ Frank Porter Graham Child Development Institute, UNC School of Medicine, The University of North Carolina at Chapel Hill, Chapel Hill, NC, United States; ^4^ Department of Epidemiology and Prevention, Department of Internal Medicine, Wake Forest University School of Medicine, Winston-Salem, NC, United States; ^5^ Departments of Psychiatry and Pediatrics and the Eunice Kennedy Shriver Center, UMass Chan Medical School, Worcester, MA, United States; ^6^ Department of Pediatrics, UMass Chan Medical School—Baystate, Springfield, MA, United States; ^7^ Department of Pediatrics, Connecticut Children’s Hospital, University of Connecticut School of Medicine, Farmington, CT, United States; ^8^ Department of Pediatrics, Wake Forest University School of Medicine, Winston-Salem, NC, United States; ^9^ Kennedy Research Center on Intellectual and Neurodevelopmental Disabilities, University of Chicago, Chicago, IL, United States

**Keywords:** social determinants of health (SDH), health-related quality of life (HRQL), neonatal follow-up, health equity (MeSH), extremely low gestation age neonates

## Abstract

**Background:**

Reducing healthcare disparities among children is extremely important given the potential impact of these disparities on long-term health-related quality of life (HRQL). Race and parental socioeconomic status (SES) are associated with child HRQL, but these associations have not been studied in infants born extremely preterm (EP), a population at increased risk for physical, cognitive, and psychosocial impairments. Achieving health equity for infants born EP across their life course requires identifying the impact of racism and SES on HRQL.

**Objective:**

We aimed to evaluate the association between self-reported maternal race, SES factors, and HRQL among 10-year-old children born EP.

**Design/methods:**

Participants were identified from an ongoing multicenter prospective longitudinal study of Extremely Low Gestational Age Newborns (ELGAN Study), born between 2002 and 2004, and evaluated at 10 years of age using the Pediatric quality of life (QoL) Inventory completed by their parent or guardian, assessing physical, emotional, social, school, and total (composite) QoL domains. Multivariable regression models were used to evaluate the relationship between QoL scores and self-identified maternal race, adjusting for SES factors (education level, marital status, and public insurance).

**Results:**

Of 1,198 study participants who were alive at 10 years of age, 863 (72.0%) were evaluated at 10 years of age. Differences in mean 10-year QoL scores across racial groups were observed and were significant on univariate analysis. However, these associations attenuated when adjusted for the marital status, public insurance status, and education status of mothers. A comparison of children with English as the primary language spoken at home vs. any other language revealed a significant difference only in school QoL, in which non-English language was associated with more favorable school QoL scores.

**Conclusions:**

Among 10-year-old children born EP, differences in parent-reported QoL were associated with maternal SES factors but not with race. Our results suggest that interventions designed to improve the SES of mothers may enhance the QoL of children born EP. Furthermore, these results underscore that race is a social construct, rather than a biological variable, as we work toward greater equity in care provision.

## Introduction

Health disparities, defined by the Centers for Disease Control, are preventable differences in the burden of disease, injury, violence, or opportunities to achieve optimal health that are experienced by socially disadvantaged populations (1). The American Academy of Pediatrics confirms that as compared to White youth, Black and Latinx youth experience disparities in health that are “extensive, pervasive, and persistent, and occur across the spectrum of health” (2). At least 43% of children younger than 18 years old report a race and ethnicity other than (non-Hispanic) White, and these groups are expected to constitute more than half of all children in the United States by 2040 (3). Disparities within children's healthcare are extremely important to address because of their potential impact on long-term health-related quality of life (HRQL) across the lifespan. Advances in neonatal–perinatal medicine over the last few decades have improved the survival rates of infants born extremely preterm (EP; less than 28 weeks gestation) (4, 5). Yet, these preterm survivors are at high risk for long-term physical, cognitive, and psychosocial impairments that can persist across the life course (5, 6). Understanding the impact of race on HRQL in this patient population may allow providers to address inequity and support patients born EP across their lifespan. Addressing healthcare disparities necessitates investigating and addressing structural racism and the social determinants of health (SDoH).

It is known that preterm infants are at increased risk of a variety of adverse developmental and health outcomes (7–11). Both during neonatal hospitalization and during childhood, racial disparities have been identified among children born preterm, with generally less favorable outcomes among Black preterm children. Similarly, it is well described that children who are Black or Latinx experience health disparities when compared with White youth across a wide spectrum of conditions although this finding is generally informed by studies focused on morbidity (e.g., obesity, asthma), mortality, and specific indicators (e.g., access to care) (2). While adverse health outcomes can impact global health, an alternative approach to disease or outcome-specific studies is to evaluate overall health through the study of quality of life (QoL) for individuals born EP. HRQL incorporates patient and caregiver perspectives regarding health status across physical, social, emotional, and school domains, and the effects of physical and social factors on QoL. HRQL aligns directly with the World Health Organization's definition of health as “the complete state of physical, mental, and social wellbeing, not merely the absence of disease” (12).

Recent work from the Healthy Passages study of elementary-age school children shows parental socioeconomic status (SES; defined using parent-reported highest level of education completed by any member of the household and total household income), as well as factors such as family cohesion, parental nurturance, other adult, and peer support, are positively associated with child QoL across all racial/ethnic categories, and when adjusted for SES status, many (but not all) HRQL differences across racial/ethnic categories become insignificant (3, 13, 14). The impact of race investigated as a proxy for racism and SDoH on HRQL in infants born EP has not been investigated. Prior studies that have evaluated SDoH in relation to cognition suggest that SDoH might mediate some, or all, of the associations between race and child outcomes (15, 16). However, none of these studies have included extremely preterm individuals, who are more likely to encounter SDoH and are more likely to exhibit adverse child health outcomes. We hypothesize that differences observed in HRQL between racial groups in the population of children born EP at age 10 years will not persist when adjusted for social factors associated with lower SES such as maternal education, marital status, Supplemental Nutrition Assistance Program (SNAP) eligibility, and public insurance status. In addition, since previous work by caregivers and patients indicates that compared with children in English-primary-language households, children in non-English-primary-language households experience worse health (17), we evaluated the hypothesis that this variable was associated with lower HRQL.

## Methods

### Cohort selection

This report follows the guidelines of the Strengthening the Reporting of Observational Studies in Epidemiology (STROBE) (18). Between 2002 and 2004, women giving birth at 28 weeks of gestation or earlier at one of 14 academic medical centers throughout the United States were enrolled in the Extremely Low Gestational Age Newborn (ELGAN) study. Maternal consent was obtained upon hospital admission or near delivery. Each participating institution received institutional review board (IRB) approval for protocols used during the study. Approximately 85% of mothers approached for participation in the original ELGAN study consented to participate, resulting in a cohort of 1,249 mothers and 1,506 infants. Of these 1,506 infants, 1,198 (80%) of those enrolled survived to 10 years of age, and 863 (72%) of this group completed a quality of life survey at 10 years of age (19). Nineteen percent of this group did not participate, and this non-participation was the result of the study design; specifically, 232 of the 1,198 surviving cohort members did not meet the following inclusion criteria for the 10-year follow-up: (1) data on inflammation-related proteins in the neonatal period and (2) data on neurodevelopmental follow-up at 24 months of age (age-adjusted for degree of prematurity). The remaining cohort members were eligible for the 10-year follow-up but could not be located by study coordinators (*n* = 77), despite attempts to reach the family using phone, email, and social media or did not complete the quality of life survey at 10 years (*n* = 26) (11).

Data were collected through maternal interviews and a review of mothers’ medical records for multiple demographic variables. These included maternal race, which was self-reported by mothers at the time of enrollment from the following: Asian, Black, Native American, White, Mixed, and Other.

Due to the multifaceted nature of SES status (20), which no single variable captures adequately, especially for racial and ethnic minorities (21, 22), we considered data on multiple contributing variables known to impact maternal financial status and opportunity and collected these data. These included maternal age category (<21, 21–35, and >35 years), maternal education (ranging from less than high school education to higher than college education), maternal marital status (including married, not ever married and living together, not ever married and not living together, and separated/divorced/widowed), maternal insurance type (public or private), SNAP eligibility (yes/no), and primary language spoken at home (English or non-English).

### Ten-year follow-up

At the 10-year follow-up visit, HRQL measures were evaluated by a parent or caregiver, who completed the Pediatric Quality of Life Inventory (PedsQL) 4.0 generic core scales (23). The PedsQL was designed to measure the core components of quality of life across multiple domains for the child—physical (eight items), emotional (five items), social (five items), and school (five items) functioning—and each item was scored on a 5-point Likert scale. These domains were summed and transformed to a linear 0–100 scale, with higher scores corresponding to a higher quality of life. The PedsQL total summary score incorporates all areas of functioning (23 items total) and provides a composite quality-of-life score for the child. The PedsQL has been demonstrated to be reliable and valid in a large population of enrollees in the Children's Health Insurance Program in California (24).

### Statistical analyses

An initial descriptive statistical analysis was completed to compare the mean values of age-10 HRQL measures among different self-identified maternal race groups. Upon noticing considerable differences among these groups, a univariate linear regression analysis was conducted to evaluate the associations between self-identified maternal race as well as QoL scores and SDoH measures, including maternal age, education, marital status, insurance type, and SNAP eligibility with QoL scores. In this analysis, groups of self-reported maternal race analyzed were White, Black, and the combined group comprising self-reported race of Asian, Native American, Mixed, and Other. Noticing the negative associations (some being statistically significant), a multivariate regression analysis was subsequently conducted considering all five factors (maternal age, education, marital status, insurance type, and SNAP eligibility) for a better understanding of the associations between maternal race and SDoH factors and child QoL at age 10 years. Statistical significance was defined as *p* < 0.05.

## Results

Of 1,198 study participants who survived until discharge, 863 (72.0%) were evaluated at 10 years. Maternal prenatal data and neonatal characteristics are summarized in Table 1. With regard to maternal race and ethnicity, 63% of our subjects self-reported as White, 26% as Black, and approximately 10% as Hispanic. Differences in mean 10-year QoL scores across racial groups were observed (Table 2) and were significant on univariate analysis, with the self-reported maternal race of Black and the combined group comprising the self-reported race of Asian, Native American, Mixed, and Other being associated with lower scores (Table 3). However, the significance of these associations was attenuated when adjusting for SDoH factors, including marital status, age, education status, public insurance status, and SNAP eligibility (Table 4). For maternal education and marital status variables, both of which consisted of five categories, a secondary set of binary variables—college education and married—was also defined to explore the associations of these factors without the small sample size effects of some categories. In the same way, the very small number of mothers whose marital status was “widowed” were analyzed with mothers “separated or divorced,” under the “separated or divorced or widowed” category. On multivariable analysis, only the SDoH factors remained significant. These included maternal higher education (more than college education), which was associated with higher HRQL scores across school and total domains. A status of separated, divorced, or widowed mother was associated with a lower school HRQL score, as compared to married or living with a partner. Public insurance (Medicaid) was associated with significantly lower HRQL scores across all domains. SNAP eligibility, another income-based national program to provide food stamp assistance to families with economic need, was associated with lower physical and total QoL scores. In addition, as compared to children with English as their primary language, those with another language as their primary language had more favorable school QoL scores (Table 5).

**Table 1:** Baseline maternal and neonatal characteristics of the overall study cohort and by sex at age 10 years.

Characteristic	Overall	Asian	Black	Mixed	Native American	Other	White
	*N* = 879	*N* = 19	*N* = 227	*N* = 25	*N* = 8	*N* = 46	*N* = 554
Ethnicity							
Non-Hispanic	800 (91%)	19 (100%)	225 (99%)	16 (64%)	3 (38%)	5 (11%)	532 (96%)
Hispanic	77 (8.8%)	0 (0%)	2 (0.9%)	9 (36%)	5 (62%)	40 (89%)	21 (3.8%)
Maternal age							
Under 21	112 (13%)	3 (16%)	45 (20%)	5 (20%)	1 (12%)	12 (26%)	46 (8.3%)
21–35	589 (67%)	14 (74%)	152 (67%)	18 (72%)	7 (88%)	30 (65%)	368 (66%)
Over 35	178 (20%)	2 (11%)	30 (13%)	2 (8.0%)	0 (0%)	4 (8.7%)	140 (25%)
Mother's education							
Less than high school	120 (14%)	3 (18%)	41 (19%)	4 (18%)	4 (50%)	15 (33%)	53 (9.8%)
High school graduate	228 (27%)	4 (24%)	82 (37%)	7 (32%)	3 (38%)	16 (36%)	116 (21%)
Some college	201 (24%)	0 (0%)	68 (31%)	7 (32%)	1 (12%)	10 (22%)	115 (21%)
College graduate	166 (19%)	5 (29%)	16 (7.3%)	2 (9.1%)	0 (0%)	4 (8.9%)	139 (26%)
More than college	139 (16%)	5 (29%)	12 (5.5%)	2 (9.1%)	0 (0%)	0 (0%)	120 (22%)
Marital status							
Married	534 (61%)	15 (79%)	67 (30%)	6 (25%)	2 (25%)	22 (48%)	422 (76%)
Separated or divorced	31 (3.5%)	0 (0%)	11 (4.8%)	3 (12%)	0 (0%)	4 (8.7%)	13 (2.3%)
Not ever married but living together	153 (17%)	4 (21%)	55 (24%)	9 (38%)	4 (50%)	12 26%)	69 (12%)
Not ever married and not living together	159 (18%)	0 (0%)	94 (41%)	6 (25%)	2 (25%)	7 (15%)	50 (9.0%)
Widowed	1 (0.1%)	0 (0%)	0 (0%)	0 (0%)	0 (0%)	1 (2.2%)	0 (0%)
Public insurance	304 (35%)	5 (29%)	140 (62%)	10 (45%)	7 (88%)	26 (58%)	116 (21%)
Food stamp eligibility	112 (13%)	0 (0%)	52 (23%)	3 (14%)	1 (12%)	14 (31%)	42 (7.7%)
Smoke while pregnant	120 (14%)	0 (0%)	32 (14%)	3 (14%)	2 (25%)	4 (8.9%)	79 (14%)
Passive smoke exposure	211 (25%)	5 (29%)	70 (32%)	6 (27%)	4 (50%)	9 (20%)	117 (21%)
Any smoke exposure	247 (29%)	5 (29%)	79 (35%)	8 (36%)	4 (50%)	12 (27%)	139 (25%)
Prepregnancy body mass index							
Healthy weight (18.5–25)	426 (50%)	10 (59%)	95 (44%)	10 (45%)	3 (38%)	15 (37%)	293 (54%)
Underweight (<18.5)	68 (8.0%)	2 (12%)	9 (4.1%)	1 (4.5%)	1 (12%)	0 (0%)	55 (10%)
Overweight (25–30)	164 (19%)	2 (12%)	50 (23%)	7 (32%)	3 (38%)	14 (34%)	88 (16%)
Obese (>30)	190 (22%)	3 (18%)	64 (29%)	4 (18%)	1 (12%)	12 (29%)	106 (20%)
Prepregnancy Asthma	103 (12%)	0 (0%)	25 (11%)	4 (18%)	0 (0%)	7 (16%)	67 (12%)
Prepregnancy Diabetes	26 (3.0%)	0 (0%)	7 (3.2%)	2 (9.1%)	0 (0%)	1 (2.2%)	16 (2.9%)
Prepregnancy hypertension	60 (7.0%)	1 (6.2%)	24 (11%)	1 (4.5%)	1 (12%)	6 (13%)	27 (5.0%)
Pregnancy toxemia	85 (9.9%)	4 (25%)	23 (10%)	2 (9.1%)	1 (12%)	7 (16%)	48 (8.8%)
Pregnancy hypertension	132 (15%)	3 (19%)	46 (21%)	2 (9.1%)	1 (12%)	11 (24%)	69 (13%)
Delivery hypertension	126 (14%)	6 (32%)	37 (16%)	3 (12%)	2 (25%)	11 (24%)	67 (12%)
Delivery HELLP	36 (4.1%)	1 (5.3%)	5 (2.2%)	1 (4.0%)	0 (0%)	2 (4.3%)	27 (4.9%)
Any hypertension	183 (21%)	6 (32%)	62 (27%)	4 (16%)	2 (25%)	13 (28%)	96 (17%)
Neonate gestational age (weeks)							
27	301 (34%)	7 (37%)	61 (27%)	12 (48%)	3 (38%)	15 (33%)	203 (37%)
25–26	393 (45%)	11 (58%)	115 (51%)	8 (32%)	5 (62%)	18 (39%)	236 (43%)
23–24	185 (21%)	1 (5.3%)	51 (22%)	5 (20%)	0 (0%)	13 (28%)	115 (21%)
Neonate sex							
Female	430 (49%)	11 (58%)	112 (49%)	10 (40%)	3 (38%)	29 (63%)	265 (48%)
Male	449 (51%)	8 (42%)	115 (51%)	15 (60%)	5 (62%)	17(37%)	289(52%)

HELLP, hemolysis, elevated liver enzymes, low platelet count.

**Table 2:** Parent-reported HRQL for 10-year-old children born extremely preterm, by self-reported maternal race.

HRQL characteristic	Asian	Black	Mixed	Native American	Other	White
	*N* = 19	*N* = 220	*N* = 25	*N* = 8	*N* = 45	*N* = 546
Physical	83.2 (18.9)	82.7 (22.4)	84.8 (21.5)	80.9 (24.3)	85.7 (15.4)	86.8 (19.4)
Emotional	78.7 (22.2)	78.3 (19.4)	75.8 (20.9)	70.0 (17.7)	75.0 (24.1)	79.0 (18.9)
Social	82.4 (25.2)	77.5 (21.3)	69.6 (25.8)	74.4 (16.8)	79.9 (23.2)	82.0 (20.5)
School	77.9 (18.4)	67.4 (20.8)	60.4 (27.7)	61.9 (16.0)	70.2 (22.1)	75.0 (20.3)
Total	80.9 (16.6)	77.3 (16.8)	74.2 (20.2)	73.0 (14.4)	78.7 (17.0)	81.5 (15.4)

Data are means (standard deviation in parentheses).

**Table 3:** Relationships between self-reported maternal race, socioeconomic factors, and five child QoL scores at 10 years of age.

	Physical summary score	Emotional functioning scale	Social functioning scale	School functioning scale	Total quality-of-life score
Maternal Characteristics	Beta (95% CI)				
Race					
White	Ref.	Ref.	Ref.	Ref.	Ref.
Black	**−4.0** **(−7.2 to −0.87)**	−0.74 (−3.8 to 2.3)	**−4.5** **(−7.8 to −1.2)**	**−7.6** **(−11 to −4.3)**	**−4.2** **(−6.7 to −1.7)**
Non-White, Non-Black	−2.2 (−6.5 to 2.2)	−3.5 (−7.7 to 0.68)	**−4.7** **(−9.3 to −0.15)**	**−6.5** **(−11 to −2.0)**	**−4** **(−7.4 to −0.48)**
Education					
Less than high school	Ref.	Ref.	Ref.	Ref.	Ref.
High school graduate	1.1 (−3.3 to 5.5)	1.4 (−2.9 to 5.7)	−0.5 (−5.2 to 4.1)	0.62 (−3.9 to 5.1)	0.70 (−2.8 to 4.2)
Some college	4.2 (−0.27 to 8.8)	2.5 (−1.9 to 7.0)	**5.1** **(0.32 to 9.8)**	**8.8** **(4.2 to 13.4)**	**5.0** **(1.5 to 8.6)**
College grad	**9.4** **(4.8 to 14.0)**	2.8 (−1.80 to 7.4)	**7.3** **(2.4 to 12.2)**	**11.8** **(7.1 to 16.6)**	**8.0** **(4.3 to 11.7)**
More than college	**10.72** **(5.83 to 15.62)**	3.96 (−0.84 to 8.76)	**8.62** **(3.46 to 13.79)**	**15.01** **(10.02 to 20.00)**	**9.73** **(5.83 to 13.63)**
Marital status					
Married	Ref.	Ref.	Ref.	Ref.	Ref.
Not ever married, living together	**−5.0** **(−8.6 to −1.4)**	−2.6 (−6.1 to 0.86)	**−5.0** **(−8.7 to −1.2)**	**−7.9** **(** **−11.5 to −4.3)**	**−5.1** **(** **−8.0 to −2.2)**
Not ever married, not living together	**−6.5** **(−10.1 to −2.9)**	−1.7 (−5.2 to 1.8)	**−4.3** **(−8.1 to −0.48)**	**−10.1** **(−13.7 to −6.4)**	**−5.8** **(−8.6 to −2.9)**
Separated/divorced/widowed	**−7.6** **(−14.8 to −0.47)**	−3.8 (−10.8 to 3.1)	−6.9 (−14.5 to 0.67)	**−16.6** **(−23.9 to −9.3)**	**−8.6** **(−14.3 to −2.9)**
Public insurance					
No	Ref.	Ref.	Ref.	Ref.	Ref.
Yes	**−8.2** **(−11.0 to −5.4)**	**−4.3** **(−7.0 to −1.5)**	**−8.3** **(−11.3 to −5.4)**	**−12.2** **(−15.1 to −9.4)**	**−8.3** **(−10.5 to −6.0)**
SNAP eligible					
No	Ref.	Ref.	Ref.	Ref.	Ref.
Yes	**−10.51** **(−14.51 to −6.52)**	**−3.90** **(−7.79 to −0.01)**	**−9.64** **(−13.85 to −5.43)**	**−12.14** **(−16.27 to −8.01)**	**−9.24** **(−12.43 to −6.05)**

SNAP, Supplemental Nutrition Assistance Program; CI, confidence interval.

Data are mean differences (95% confidence intervals in parentheses) estimated using unadjusted linear regression models. The bold font indicates that associations are statistically significant with a *p*-value < 0.05.

**Table 4:** Relationships between self-reported maternal race, socioeconomic factors, and five child QoL scores at 10 years of age.

	Physical summary score	Emotional functioning scale	Social functioning scale	School functioning scale	Total quality-of-life score
*Maternal Characteristics*	Beta (95% CI)				
Race					
White	Ref.	Ref.	Ref.	Ref.	Ref.
Black	0.03 (−3.6 to 3.6)	0.36 (−3.2 to 3.9)	−1.8 (−5.6 to 1.9)	−2.5 (−6.1 to 1.1)	−0.85 (−3.7 to 2.0)
Non-White, Non-Black	0.89 (−3.7 to 5.5)	−2.6 (−7.1 to 1.9)	−1.9 (−6.8 to 2.9)	−1.5 (−6.2 to 3.1)	−1 (−4.6 to 2.6)
Education					
Less than high school	Ref.	Ref.	Ref.	Ref.	Ref.
High school graduate	−0.19 (−4.8 to 4.4)	0.64 (−3.9 to 5.2)	−1.2 (−6.1 to 3.6)	−0.78 (−5.4 to 3.9)	−0.36 (−4.0 to 3.3)
Some college	1 (−3.9 to 6.0)	0.24 (−4.6 to 5.1)	2.2 (−3.0 to 7.4)	4.7 (−0.33 to 9.6)	1.9 (−2.0 to 5.8)
College grad	4.9 (−0.55 to 10)	−0.15 (−5.5 to 5.2)	2.8 (−3.0 to 8.6)	4.9 (−0.60 to 10)	3.4 (−0.96 to 7.7)
More than college	5.7 (−0.03 to 11)	0.55 (−5.1 to 6.2)	3.7 (−2.4 to 9.7)	**7.6** **(1.7 to 13)**	**4.5** **(0.01 to 9.1)**
Marital status					
Married	Ref.	Ref.	Ref.	Ref.	Ref.
Not ever married, living together	0.86 (−3.5 to 5.2)	−0.93 (−5.2 to 3.3)	0.47 (−4.1 to 5.0)	−1.1 (−5.5 to 3.3)	−0.04 (−3.5 to 3.4)
Not ever married, not living together	−0.32 (−4.9 to 4.2)	1.5 (−3.0 to 6.0)	2.9 (−1.9 to 7.7)	−1.5 (−6.1 to 3.1)	0.53 (−3.1 to 4.1)
Separated, divorced, or widowed	−0.87 (−8.7 to 6.9)	−0.01 (−7.7 to 7.7)	−0.25 (−8.5 to 8.0)	**−8.9** **(−17 to −1.0)**	−2.3 (−8.5 to 3.9)
Public insurance					
No	Ref.	Ref.	Ref.	Ref.	Ref.
Yes	**−4.7** **(−8.6 to −0.71)**	**−4.3** **(−8.2 to −0.36)**	**−6.1** **(−10 to −1.9)**	**−6.1** **(−10 to −2.0)**	**−5.2** **(−8.3 to −2.1)**
SNAP eligible					
No	Ref.	Ref.	Ref.	Ref.	Ref.
Yes	**−5.8** **(−10 to −1.3)**	−1.4 (−5.8 to 3.1)	−4.4 (−9.1 to 0.37)	−3.5 (−8.1 to 1.0)	**−4** **(7.6 to −0.46)**

SNAP, Supplemental Nutrition Assistance Program; CI, confidence interval.

Data are mean differences between QoL scores (95% confidence intervals in parentheses) estimated using a multivariate linear regression model. The bold font indicates that associations are statistically significant with a *p*-value < 0.05.

**Table 5:** A comparison of HRQL measures between children with English as their primary language and those with another language as their primary language.

Characteristic	English	Non-English	*p*-value
	(*N* = 760)	(*N* = 126)	
Physical	85.2 (20.6)	86.4 (17.8)	0.8
Emotional	78.2 (19.3)	79.7 (20.6)	0.2
Social	79.9 (21.3)	83.3 (20.5)	0.069
School	**71.7 (21.1)**	**76.0 (19.7)**	**0.041**
Total	79.6 (16.3)	82.0 (15.3)	0.11

Data are mean (standard deviation). The *p*-value is based on the Wilcoxon rank sum test.

The bold values indicate the statistical significant *p-*value (<0.05).

## Discussion

We found that once adjusted for SES factors, the association between race and HRQL in children born EP at 10 years was greatly attenuated and no longer statistically significant. Given that both race and ethnicity are more of a social construct rather than a truly biological variable, our findings highlight the fact that racial disparities in quality-of-life outcomes are likely explained by the social, economic, and healthcare resources available to racial and ethnic minorities in the United States. As hypothesized, lower SES factors (reliance on public insurance, SNAP eligibility, mother separated, divorced, or widowed status) were correlated with lower child QoL scores on subscores (physical, emotional, social, school) as well as impact some composite (total QoL) domains. Predictably, advanced maternal education (more than college) was associated with a higher school QoL score and a higher total QoL score. While other associations remained non-significant, we observed a dose-dependent relationship between increasing maternal education and higher school QoL scores, consistent with that of previous literature, which supports and strengthens the positive impact of maternal education on child health (25, 26).

SNAP eligibility, an income-based program providing assistance primarily with food and also other necessary products for daily living, was associated with a lower physical QoL score and a lower total QoL score. This finding is consistent with that of prior work identifying associations between food insecurity and multiple adverse physical health outcomes in children, including increased rates of asthma, lower utilization of preventative medical care, and higher emergency department usage (27). Reliance on public insurance, an indicator of lower household income, was associated with lower QoL scores across all domains. This finding is consistent with findings from the Healthy Passages Study, in which lower child HRQL was associated with parental SES and other SDoH factors, and when adjusted for these social factors, race was not associated with child HRQL (3, 13, 14). Thus, the current study agrees with the findings of others that racial/ethnic minority status is frequently associated with lower SES, which, in turn, is associated with suboptimal health outcomes in children (20, 28–31).

Previous work indicates that compared with children in English-primary-language households, children in non-English-primary-language households experience worse health and are more likely to be poor, uninsured or sporadically insured, and have no usual source of medical care (32–34). In our cohort, children in households with another language besides English had comparable HRQL scores that were no different from those for children in English-primary-language households. Importantly, we were surprised to find that non-English-speaking children had higher school HRQL. As we have tried to demonstrate that SDoH and low SES are associated with lower HRQL, it may have been expected that HRQL might be lower when children experience these social and economic adversities. One explanation for this finding may be a nuanced difference between the primary language spoken at home and limited English proficiency (LEP). LEP (the parents’ self-reported ability to speak English very well, well, not very well, or not at all) has been shown to exhibit a dose-dependent relationship between children's insurance coverage, parental educational attainment, and family income. Lower LEP is associated with less insurance and parent education and lower income, while primary language spoken at home is not as strongly associated (17). Our findings may be capturing the primary language spoken at home as a variable of cultural practice and parental choice and not parental LEP. It is also plausible that for this subset of families, although resource-challenged, as first-generation immigrants, their families are highly motivated to support their children's wellbeing and educational achievements for the betterment of their future. Further investigation is needed to disentangle these relationships, but our data indicate that language at home is probably not driving differences in HRQL observed in children born EP at age 10 years.

This study has implications for clinical care and future healthcare policy and research. Disparities in child HRQL in a patient population born EP among racial groups are associated with SDoH variables, and focusing on disparities in these variables may provide insights into mitigating racial disparities. Addressing socioeconomic disparities alone is not sufficient, as it does not correct the systems of oppression that underlie these disparities. Even among women of high SES—college education, private insurance, not receiving Women, Infants, and Children (WIC) benefits—disparities in preterm birth rates persist, with non-Hispanic Black women having increased rates of premature birth than non-Hispanic White women (35). Racial, ethnic, and socioeconomic disparities remain widespread in the United States, and addressing these disparities is necessary for providing safe and equitable care. Yet, improving access to healthcare *can* improve child HRQL, as demonstrated by the success of the California State Children's Health Insurance Program and the impact of the Earned Income Tax Credit on infant health (36–38). Disparities in child health are implicated in large social and financial costs across the life span (39), and optimizing child health is critical for maximizing health throughout life (40). Among this population of infants born EP, early and timely identification of at-risk mother–infant dyads who may benefit from comprehensive biopsychosocial interventions has the potential to improve patient HRQL well beyond the neonatal period (41). These focused interventions with postbirth hospitalization discharge home visits by nurses and social workers have been found to reduce rates of infant mortality and rehospitalization following initial discharge among preterm infants (42). Education and support for infant stimulation initiated in the hospital and continued at home have also been shown to achieve the social interaction patterns essential for optimal development in a cohort of infants born prematurely with mothers with social–environmental risk factors (43). Furthermore, the benefits of early intervention services among children born prematurely are well-documented, with demonstrated benefits observed throughout childhood and into adulthood (44–46). Addressing the financial insecurity of mothers caring for a vulnerable child is a necessity, with further research and implementation needed. A randomized control trial of 46 Medicaid-eligible mothers of preterm infants found that financial support (up to 3 weekly financial transfers of $200 each their infant was hospitalized) increased skin-to-skin care and breast milk provision, indicating the role of financial support to facilitate mothers’ engagement with caregiving behaviors (47).

The strengths of our study include a relatively high follow-up rate of a large, prospective, multicenter cohort sample that was diverse with respect to sociodemographic attributes and geographic location, with patients enrolled from 14 institutions across central and eastern United States. There also are several limitations. The observational design inherent to the study prevented us from directly investigating the causes of racial disparities. We lacked a sufficient sample size to address Latinx ethnicity within our study and recognize the extensive body of literature documenting healthcare disparities afflicting this patient population (2). Only EP individuals were included in the ELGAN Study, and therefore, the findings reported here might not apply to children born preterm beyond 28 weeks’ gestation and closer to term. Lastly, 28% of the potential study sample was not evaluated at 10 years of age, potentially resulting in selection bias.

## Conclusions

Among 10-year-old children born EP, differences in parent-reported QoL were associated with maternal SES factors but not with race. Our results suggest that interventions designed to improve mothers’ SES may enhance the QoL of children born EP. Furthermore, these results underscore that race is a social construct, rather than a biological variable, as we work toward greater equity in care provision.

## Data Availability

All data are available by completing the NINDS Data Request Form, which is available from Archived Clinical Research Datasets and sending the completed form to CRLiaison@ninds.nih.gov.

## ELGAN-ECHO Study Investigators

Project Lead for ELGAN-2 & ELGAN ECHO: Julie V. Rollins, MA.

### Site Principal Investigators

Baystate Medical Center, Springfield, MA: Bhavesh Shah, MD; Rachana Singh, MD, MS; Ruben Vaidya, MD.

Boston Children’s Hospital, Boston, MA: Linda Van Marter, MD, MPH; Camilla Martin, MD, MPH; Janice Ware, PhD; Caitlin Rollins, MD.

Tufts Medical Center, Boston, MA: Cynthia Cole, MD; Ellen Perrin, MD; Christina Sakai.

University of Massachusetts Medical School, Worcester, MA: Frank Bednarek, MD (deceased); Jean Frazier, MD.

Yale University School of Medicine, New Haven, CT: Richard Ehrenkranz, MD (deceased); Jennifer Benjamin, MD; Angela Montgomery, MD.

Wake Forest University, Winston-Salem, NC: T. Michael O’Shea, MD, MPH; Lisa Washburn, MD; Semsa Gogcu, MD, MPH.

University of North Carolina at Chapel Hill, Chapel Hill, NC: Carl Bose, MD; Diane Warner, MD, MPH; T. Michael O’Shea, MD, MPH.

East Carolina University, Greenville, NC: Steve Engelke, MD; Amanda Higginson, MD; Jason Higginson, MD; Kelly Bear, MD.

Helen DeVos Children’s Hospital, Grand Rapids, MI: Mariel Poortenga, MD; Steve Pastyrnak, PhD.

Sparrow Hospital, Lansing, MI, and Michigan State University, East Lansing, MI; Padu Karna, MD; Nigel Paneth, MD, MPH; Madeleine Lenski, MSPH.

University of Chicago Medical Center, Chicago, IL: Michael Schreiber, MD; Scott Hunter, PhD; Michael Msall, MC.

William Beaumont Hospital, Royal Oak, MI: Danny Batton, MD; Judith Klarr, MD; Young Ah Lee, MD; Rawad Obeid, MD.

### Site Study Coordinators

Baystate Medical Center, Springfield, MA: Karen Christianson, RN; Deborah Klein, BSN, RN; Katie Wagner, MS; Victoria Cobb; Shaula Paula; Andres Santana.

Boston Children’s Hospital, Boston, MA: Maureen Pimental, BA; Collen Hallisey, BA; Taryn Coster, BA; Maddie Dolins; Maggie Mittleman; Hannah Haile; Julia Rohde; Kaysi Herrera Pujols; Susie Rodriquez; Kyla Waring.

Tufts Medical Center, Boston, MA: Ellen Nylen, RN; Emily Neger, MA; Kathryn Mattern, BA; Catherine Ma; Deanna Toner; Elizabeth Vitaro; Allison Nolan.

University of Massachusetts Medical School, Worcester, MA: Lauren Venuti, BA; Beth Powers, RN; Ann Foley, EdM; Taylor Merk.

Yale University School of Medicine, New Haven, CT: Joanne Williams, RN; Elaine Romano, APRN; Christine Henry.

Wake Forest University, Winston-Salem, NC: Debbie Hiatt, BSN (deceased); Nancy Peters, RN; Patricia Brown, RN; Emily Ansusinha, BA; Jazmyne James, MS; Nou Yang, MS; Nicole Froelich; Kristi Lanier.

University of North Carolina at Chapel Hill, Chapel Hill, NC: Gennie Bose, RN; Janice Wereszczak, MSN; Janice Bernhardt, MS, RN.

East Carolina University, Greenville, NC: Joan Adams (deceased); Donna Wilson, BA, BSW; Nancy Darden-Saad, BS, RN; Bree Williams; Emily Jones; Hannah Morris; Taiara Williams; Isabella Carter; Emily Jones.

Helen DeVos Children’s Hospital, Grand Rapids, MI: Dinah Sutton, RN; Julie Rathbun, BSW, BSN; Stephanie Fagerman; William Boshoven; Jalen Johnson; Brandon James; Cynthia Gile, BS, CCRC; Megan Maynard; Emina Nakic; Cynthia Gile; Duvonna Haynes.

Sparrow Hospital, Lansing, MI, and Michigan State University, East Lansing, MI: Karen Miras, RN, BSN; Carolyn Solomon, RN; Deborah Weiland, MSN; Chloe Caltrider.

University of Chicago Medical Center, Chicago, IL: Grace Yoon, RN; Rugile Ramoskaite, BA; Suzanne Wiggins, MA; Krissy Washington, MA; Ryan Martin, MA; Barbara Prendergast, BSN, RN; Emma Lynch, MPH; Sabina Hajdarovic.

William Beaumont Hospital, Royal Oak, MI: Beth Kring, RN.

### Psychologists

Baystate Medical Center, Springfield, MA: Anne Smith, PhD; Susan McQuiston, PhD.

Boston Children’s Hospital, Boston, MA: Samantha Butler, PhD; Rachel Wilson, PhD; Kirsten McGhee, PhD; Patricia Lee, PhD; Aimee Asgarian, PhD; Anjali Sadhwani, PhD; Brandi Henson, PsyD.

Tufts Medical Center, Boston, MA: Cecelia Keller, PT, MHA; Jenifer Walkowiak, PhD; Susan Barron, PhD.

University of Massachusetts Medical School, Worcester, MA: Alice Miller, PT, MS; Brian Dessureau, PhD; Molly Wood, PhD; Jill Damon-Minow, PhD.

Yale University School of Medicine, New Haven, CT: Elaine Romano, MSN; Linda Mayes, PhD; Kathy Tsatsanis, PhD; Katarzyna Chawarska, PhD; Sophy Kim, PhD; Susan Dieterich, PhD; Karen Bearrs, PhD.

Wake Forest University Baptist Medical Center, Winston-Salem, NC: Ellen Waldrep, MA; Jackie Friedman, PhD; Gail Hounshell, PhD; Debbie Allred, PhD.

University Health Systems of Eastern Carolina, Greenville, NC: Rebecca Helms, PhD; Lynn Whitley, PhD; Gary Stainback, PhD.

University of North Carolina at Chapel Hill, Chapel Hill, NC: Lisa Bostic, OTR/L; Amanda Jacobson, PT; Joni McKeeman, PhD; Echo Meyer, PhD.

Helen DeVos Children's Hospital, Grand Rapids, MI: Steve Pastyrnak, PhD.

Sparrow Hospital, Lansing, MI, and Michigan State University, East Lansing, MI: Joan Price, EdS; Megan Lloyd, MA, EdS.

University of Chicago Medical Center, Chicago, IL: Susan Plesha-Troyke, OT; Megan Scott, PhD.

William Beaumont Hospital, Royal Oak, MI: Katherine M. Solomon, PhD; Kara Brooklier, PhD; Kelly Vogt, PhD.

## References

[1] Centers for Disease Control and Prevention DoAaSH. Health Disparities (2023). Available online at: https://www.cdc.gov/healthyyouth/disparities/index.htm (accessed December 10, 2023).

[2] Flores G, Research CoP. Racial and ethnic disparities in the health and health care of children. Pediatrics. (2010) 125(4):e979–1020. 10.1542/peds.2010-0188 20351000

[3] Wallander JL Fradkin C Chien AT Mrug S Banspach SW Racial/ethnic disparities in health-related quality of life and health in children are largely mediated by family contextual differences. Acad Pediatr. (2012) 12(6):532–8. 10.1016/j.acap.2012.04.005 22884796 PMC12991320

[4] Stoll BJ Hansen NI Bell EF Walsh MC Carlo WA Shankaran S Trends in care practices, morbidity, and mortality of extremely preterm neonates, 1993–2012. JAMA. (2015) 314(10):1039–51. 10.1001/jama.2015.10244 26348753 PMC4787615

[5] Saigal S Doyle LW. An overview of mortality and sequelae of preterm birth from infancy to adulthood. Lancet. (2008) 371(9608):261–9. 10.1016/S0140-6736(08)60136-1 18207020

[6] Wolke D Johnson S Mendonça M. The life course consequences of very preterm birth. Annu Rev Dev Psychol. (2019) 1:69–92. 10.1146/annurev-devpsych-121318-084804

[7] Kaufman AS Kaufman NL. K-BIT 2: Kaufman Brief Intelligence Test. San Antonio, Texas: Pearson (2004).

[8] Yudkin P Aboualfa M Eyre J Redman C Wilkinson A. New birthweight and head circumference centiles for gestational ages 24 to 42 weeks. Early Hum Dev. (1987) 15(1):45–52. 10.1016/0378-3782(87)90099-5 3816638

[9] Leviton A Paneth N Reuss ML Susser M Allred EN Dammann O Maternal infection, fetal inflammatory response, and brain damage in very low birth weight infants. Pediatr Res. (1999) 46(5):566. 10.1203/00006450-199911000-00013 10541320

[10] Douglass LM Heeren TC Stafstrom CE DeBassio W Allred EN Leviton A Cumulative incidence of seizures and epilepsy in ten-year-old children born before 28 weeks’ gestation. Pediatr Neurol. (2017) 73:13–9. 10.1016/j.pediatrneurol.2017.05.009 28619377 PMC5524375

[11] Kuban KC Joseph RM O’Shea TM Allred EN Heeren T Douglass L Girls and boys born before 28 weeks gestation: risks of cognitive, behavioral, and neurologic outcomes at age 10 years. J Pediatr. (2016) 173:69–75.e1. 10.1016/j.jpeds.2016.02.048 27004675 PMC4884461

[12] World Health Organization. Constitution of the World Health Organization (2023). Available online at: https://www.who.int/about/accountability/governance/constitution (accessed December 10, 2023).

[13] Wallander JL Fradkin C Elliott MN Cuccaro PM Tortolero Emery S Schuster MA. Racial/ethnic disparities in health-related quality of life and health status across pre-, early-, and mid-adolescence: a prospective cohort study. Qual Life Res. (2019) 28:1761–71. 10.1007/s11136-019-02157-1 30927145

[14] Scott SM Wallander JL Elliott MN Grubaum JA Chien AT Tortolero S Do social resources protect against lower quality of life among diverse young adolescents? J Early Adolesc. (2016) 36(6):754–82. 10.1177/0272431615588367

[15] LeWinn KZ Bush NR Batra A Tylavsky F Rehkopf D. Identification of modifiable social and behavioral factors associated with childhood cognitive performance. JAMA Pediatr. (2020) 174(11):1063–72. 10.1001/jamapediatrics.2020.2904 32955555 PMC7506587

[16] Lawlor DA Najman JM Batty GD O'Callaghan MJ Williams GM Bor W. Early life predictors of childhood intelligence: findings from the mater-university study of pregnancy and its outcomes. Paediatr Perinat Epidemiol. (2006) 20(2):148–62. 10.1111/j.1365-3016.2006.00704.x 16466433

[17] Flores G Abreu M Tomany-Korman SC. Limited English proficiency, primary language at home, and disparities in children’s health care: how language barriers are measured matters. Public Health Rep. (2005) 120(4):418–30. 10.1177/003335490512000409 16025722 PMC1497749

[18] Von Elm E Altman DG Egger M Pocock SJ Gøtzsche PC Vandenbroucke JP. The strengthening the reporting of observational studies in epidemiology (STROBE) statement: guidelines for reporting observational studies. Lancet. (2007) 370(9596):1453–7. 10.1016/S0140-6736(07)61602-X 18064739

[19] O'Shea TM Allred EN Dammann O Hirtz D Kuban KC Paneth N The ELGAN study of the brain and related disorders in extremely low gestational age newborns. Early Hum Dev. (2009) 85(11):719–25. 10.1016/j.earlhumdev.2009.08.060 19765918 PMC2801579

[20] Chen E Martin AD Matthews KA. Understanding health disparities: the role of race and socioeconomic status in children’s health. Am J Public Health. (2006) 96(4):702–8. 10.2105/AJPH.2004.048124 16507739 PMC1470539

[21] Kaufman JS Cooper RS McGee DL. Socioeconomic status and health in blacks and whites: the problem of residual confounding and the resiliency of race. Epidemiology. (1997) 8(6):621–8. 9345660

[22] Williams DR. Race, socioeconomic status, and health the added effects of racism and discrimination. Ann N Y Acad Sci. (1999) 896(1):173–88. 10.1111/j.1749-6632.1999.tb08114.x 10681897

[23] Varni JW Burwinkle TM Seid M Skarr D. The PedsQL™* 4.0 as a pediatric population health measure: feasibility, reliability, and validity. Ambul Pediatr. (2003) 3(6):329–41. 10.1367/1539-4409(2003)003<0329:TPAAPP>2.0.CO;2 14616041

[24] Douglass LM Kuban K Tarquinio D Schraga L Jonas R Heeren T A novel parent questionnaire for the detection of seizures in children. Pediatr Neurol. (2016) 54:64–9.e1. 10.1016/j.pediatrneurol.2015.09.016 26552646

[25] Sirin SR. Socioeconomic status and academic achievement: a meta-analytic review of research. Rev Educ Res. (2005) 75(3):417–53. 10.3102/00346543075003417

[26] Reardon SF. The widening academic achievement gap between the rich and the poor: new evidence and possible explanations. Whither Oppor. (2011) 1(1):91–116.

[27] Thomas M Miller DP Morrissey TW. Food insecurity and child health. Pediatrics. (2019) 144(4):e20190397. 10.1542/peds.2019-0397 31501236

[28] Rajmil L Herdman M Ravens-Sieberer U Erhart M Alonso J Group EK. Socioeconomic inequalities in mental health and health-related quality of life (HRQOL) in children and adolescents from 11 European countries. Int J Public Health. (2014) 59:95–105. 10.1007/s00038-013-0479-9 23793782

[29] Xiang L Su Z Liu Y Huang Y Zhang X Li S Zhang H. Impact of family socioeconomic status on health-related quality of life in children with critical congenital heart disease. J Am Heart Assoc. (2019) 8(1):e010616. 10.1161/JAHA.118.010616 30563422 PMC6405710

[30] Zeller MH Modi AC. Predictors of health-related quality of life in obese youth. Obesity. (2006) 14(1):122–30. 10.1038/oby.2006.15 16493130

[31] Aspesberro F Mangione-Smith R Zimmerman JJ. Health-related quality of life following pediatric critical illness. Intensive Care Med. (2015) 41:1235–46. 10.1007/s00134-015-3780-7 25851391

[32] Flores G Tomany-Korman SC. The language spoken at home and disparities in medical and dental health, access to care, and use of services in US children. Pediatrics. (2008) 121(6):e1703–14. 10.1542/peds.2007-2906 18519474

[33] Kirkman-Liff B Mondragón D. Language of interview: relevance for research of Southwest Hispanics. Am J Public Health. (1991) 81(11):1399–404. 10.2105/AJPH.81.11.1399 1951794 PMC1405679

[34] Weinick RM Krauss NA. Racial/ethnic differences in children’s access to care. Am J Public Health. (2000) 90(11):1771. 10.2105/AJPH.90.11.1771 11076248 PMC1446405

[35] Johnson JD Green CA Vladutiu CJ Manuck TA. Racial disparities in prematurity persist among women of high socioeconomic status. Am J Obstet Gynecol MFM. (2020) 2(3):100104. 10.1016/j.ajogmf.2020.100104 33179010 PMC7654959

[36] Seid M Varni JW Cummings L Schonlau M. The impact of realized access to care on health-related quality of life: a two-year prospective cohort study of children in the California State Children’s Health Insurance Program. J Pediatr. (2006) 149(3):354–61. 10.1016/j.jpeds.2006.04.024 16939746

[37] Hoynes H Miller D Simon D. Income, the earned income tax credit, and infant health. Am Econ J. (2015) 7(1):172–211. 10.1257/pol.20120179

[38] Hamad R Rehkopf DH. Poverty, pregnancy, and birth outcomes: a study of the earned income tax credit. Paediatr Perinat Epidemiol. (2015) 29(5):444–52. 10.1111/ppe.12211 26212041 PMC4536129

[39] Von Rueden U Gosch A Rajmil L Bisegger C Ravens-Sieberer U. Socioeconomic determinants of health related quality of life in childhood and adolescence: results from a European study. J Epidemiol Community Health. (2006) 60(2):130. 10.1136/jech.2005.039792 16415261 PMC2566139

[40] Harter S. Developmental perspectives on the self system. HandbChild Psychol. (1983) 4:275–385.

[41] Ramey CT. The abecedarian approach to social, educational, and health disparities. Clin Child Fam Psychol Rev. (2018) 21:527–44. 10.1007/s10567-018-0260-y 29637322

[42] Casey PH Irby C Withers S Dorsey S Li J Rettiganti M. Home visiting and the health of preterm infants. Clin Pediatr (Phila). (2017) 56(9):828–37. 10.1177/0009922817715949 28720035

[43] White-Traut R Norr KF Fabiyi C Rankin KM Li Z Liu L. Mother–infant interaction improves with a developmental intervention for mother–preterm infant dyads. Infant Behav Dev. (2013) 36(4):694–706. 10.1016/j.infbeh.2013.07.004 23962543 PMC3858517

[44] McCormick MC McCarton C Tonascia J Brooks-Gunn J. Early educational intervention for very low birth weight infants: results from the infant health and development program. J Pediatr. (1993) 123(4):527–33. 10.1016/S0022-3476(05)80945-X 7692028

[45] Litt JS Glymour MM Hauser-Cram P Hehir T McCormick MC. Early intervention services improve school-age functional outcome among neonatal intensive care unit graduates. Acad Pediatr. (2018) 18(4):468–74. 10.1016/j.acap.2017.07.011 28780329

[46] McCormick MC Brooks-Gunn J Buka SL Goldman J Yu J Salganik M Early intervention in low birth weight premature infants: results at 18 years of age for the infant health and development program. Pediatrics. (2006) 117(3):771–80. 10.1542/peds.2005-1316 16510657

[47] Andrews KG Martin MW Shenberger E Pereira S Fink G McConnell M. Financial support to Medicaid-eligible mothers increases caregiving for preterm infants. Matern Child Health J. (2020) 24:587–600. 10.1007/s10995-020-02905-7 32277384

